# Analysis of bacterial biodiversity and ecological risk evaluation of organophosphorus in Lianhuan Lake

**DOI:** 10.1371/journal.pone.0332712

**Published:** 2025-09-26

**Authors:** Shang Yang, Ran Wang, Wei Zhao

**Affiliations:** 1 College of Heilongjiang river and lake chief, Heilongjiang University, Harbin, P.R. China; 2 State Key Laboratory of Urban Water Resource and Environment, Harbin Institute of Technology, Harbin , P. R. China; VIT University, INDIA

## Abstract

Shallow lakes are vital water resources, playing key roles in water conservation, landscape management, and drinking water supply. However, recent years have seen significant challenges in their ecological protection. Lianhuan Lake is selected as the study area. Water quality characteristics are evaluated using the eutrophication index (TLI), water quality index (WQI) and integrated water quality index (IWQI). High-throughput sequencing (HTS) is employed to analyze microbial diversity and community composition, while gas chromatography (GC) was used to identify commonly used organophosphorus pesticides—including suprofos, coumaphos, prothiophos and mevinphos. Ecological Structure-Activity Relationships (ECOSAR), the Toxicity Estimation Software Tool (T.E.S.T.) and the Risk Quotient (RQ) are used to evaluate the ecological safety of Lianhuan Lake. The results indicate that the water quality of the Lianhuan Lake is class V, mildly eutrophic. A total of 1,062 microbial genera are identified in the sediments and water samples. The order of influence of water quality indicators on bacterial diversity is pH > TP > DO > TN > NH3-N > NO2-N > WT. Ten distinct organic phosphorus contaminants are detected. Nine of these compounds exhibited varying degrees of acute and chronic toxicity to fish, daphnia, and algae. Suprofos, coumaphos and prothiophos are highly toxic to daphnia. Suprofos, mevinphos and prothiophos have high ecological risk. These findings not only provide critical insights for the targeted management and pollution control of Lianhuan Lake but also underscore the need for monitoring organophosphorus pesticide residues in shallow lakes worldwide to protect aquatic ecosystems.

## 1. Introduction

Shallow lakes are highly biodiverse ecosystems that play essential roles in ecological processes [1]. They also provide vital habitats for numerous organisms [2]. However, with the expansion of industry, agriculture, and socio-economic development, the use of pesticides and fertilizers has increased, alongside the growing discharge of residential sewage, resulting in significant water pollution [3]. Eutrophication of lakes has become a major global environmental issue [4]. Extensive research has been conducted on this topic. For example, Zhu et al. examined the water quality and phytoplankton dynamics in Chaohu Lake, finding it to be moderately eutrophic [5]. Similarly, Suresh et al. highlighted that eutrophication is characterized by elevated nitrogen (N) and phosphorus (P) levels in water bodies, which promote the growth of harmful algae and phytoplankton biomass [6]. Zhou et al. demonstrated that biological treatment is a widely adopted method for mitigating eutrophication [7]. Recent years have seen some improvement in the management of lake eutrophication.

Lianhuan Lake, located in Dulbert Mongolian Autonomous County, Daqing City, Heilongjiang Province, China, is a large shallow lake situated on the Songnen Plain. The lake consists of 18 smaller bodies of water, including Habuta Pao, Talahong Pao, Xihulu Pao, Hongyuan Pao, and East Lake, covering a total area of over 840 square kilometers. Each year, Lianhuan Lake receives 120 million cubic meters of water from the Nenjiang River. It serves as a crucial water source for towns, agriculture, fisheries, and wetland ecosystems in Daqing City. Despite its significance, research on the biodiversity and ecological security of Lianhuan Lake has been limited in recent years.

To gain a comprehensive understanding of the biological condition of lakes and provide a scientific foundation for their management and conservation, various assessment methodologies, such as the WQI and the TLI, have been employed [8]. WQI is a metric used to assess the overall water quality by evaluating specific water quality parameters [9], and it is a common tool for analyzing pollution levels and identifying patterns in water quality [10]. El-Serehy et al. review notes that the WQI “reduces big amounts of data to a single number in a simple reproducible manner,” making it useful to managers and policymakers [11]. However, it is important to note that there is no universally accepted method for calculating the WQI [12]. Each index has its own advantages and limitations, which may constrain its applicability and ease of use. To enhance the assessment of water contamination risks in Lianhuan Lake, we applied the innovative IWQI (Improved Water Quality Index) methodology [13]. Fu et al. proposed an improved IWQI that considers both lower and upper thresholds for water quality parameters, which was applied to the assessment of the Tuojiang River Basin in Sichuan Province [14]. TLI is used to assess the trophic status of lakes and predict future trends under environmental changes [15]. Biodiversity analysis typically relies on high-throughput sequencing techniques, which offer rapid detection and high precision [16]. For example, Buccheri et al. used high-throughput sequencing of 16S and 18S rRNA genes to investigate the Mediterranean tourist site of Aci Castello [17]. Zhao et al. highlighted that high-throughput sequencing enables swift and precise identification of harmful microorganisms in aquatic systems, overcoming the challenges posed by complex environmental samples and low concentrations [18]. High-throughput sequencing is increasingly used to identify intricate community structures and biodiversity [19,20].

Many studies on ecological security evaluation employ methods such as hierarchical analysis [21], neural networks [22], and others. However, these methods often fail to accurately identify potentially toxic groups in target compounds. The ECOSAR program [23], a computer-based prediction tool, assesses the toxicity of substances to aquatic organisms by using Structure-Activity Relationships (SARs) to evaluate both acute (short-term) and chronic (long-term) toxicity to fish, aquatic invertebrates, and plants. Massarsky et al. critically evaluated ECOSAR for predicting per- and polyfluoroalkyl substances (PFAS) ecological risks, highlighting strengths and limitations across chemical classes [24]. The T.E.S.T. software [25] can process large datasets and accurately identify toxic groups within target compounds, enabling rapid toxicity assessments for chemicals with similar structures. Furthermore, it can comprehensively predict the toxicity of the target substances. Zhou et al. evaluated seven in silico tools—including ECOSAR and T.E.S.T.—against 37 Priority Controlled Chemicals and 92 New Chemicals in China for Daphnia and fish acute toxicity prediction [26]. RQ analysis [27] is a comprehensive method used to evaluate pollutant exposure and hazard effects. Montuori et al. conducted a seasonal survey in the Sele River Estuary (Southern Italy), reporting that worst‐case RQ values for chlorpyrifos, pyrimiphos‐methyl, and parathion exceeded 1.0, underscoring the need for targeted monitoring [28]. Currently, these three approaches are widely used to assess water quality safety. The combined use of ECOSAR, T.E.S.T., and RQ provides a more effective ecological safety assessment compared to traditional detection methods.

To enable the assessment of the water quality characteristics, microbial diversity, and ecological security of the Lianhuan Lake, various analytical methods are employed. Chemical analysis methods are used to analyze the physical and chemical indicators, the TLI is used to assess the lake’s eutrophication, the WQI and IWQI are used to evaluate the water quality, high-throughput sequencing technology is utilized to analyze the biodiversity of the Lianhuan Lake, gas chromatography is employed to analyze organophosphorus in water and sediment, and ECOSAR, T.E.S.T, and RQ evaluation methods are adopted to assess the ecological safety of the Lianhuan Lake. It provides data support for the evaluation index system of organic phosphorus pollution in shallow lakes and the selection of evaluation methods, and to provide decision-making basis for maintaining the ecological stability of lakes and reservoirs.

## 2. Materials and methods

### 2.1 Materials

**Fig 1:**
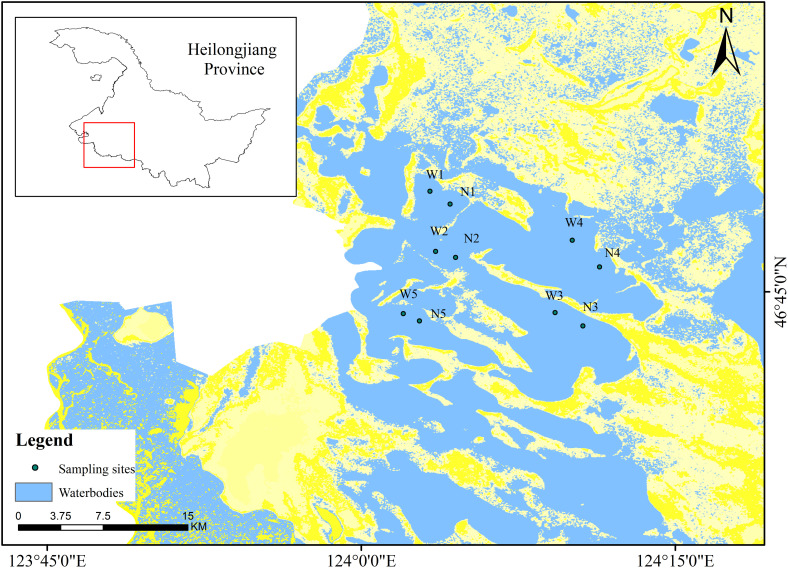
Location of the study area. Map generated by the authors using Natural Earth (public domain; https://www.naturalearthdata.com/) and HydroLAKES (public domain; https://www.hydrosheds.org/page/hydrolakes) datasets, combined with SRTM DEM hillshade.

The specimens are gathered at Lianhuan Lake in Daqing City, located in Heilongjiang Province (as shown in Fig 1). The sample location and sampling sites are shown in Table 1. All sampling sites are public waters managed by the Daqing Water Conservancy and Ecology Bureau and do not require special sampling permits. This study did not involve protected species or private land.

**Table 1:** Sample location in Lianhuan Lake.

Sampling position	Sample Number	Longitude	Latitude
DL	N1	124.071237	46.761559
	W1	124.070886	46.811154
YCS	N2	124.069657	46.746471
	W2	124.066706	46.795416
XHL2	N3	124.112458	46.741678
	W3	124.108784	46.790489
TLH	N4	124.158351	46.684257
	W4	124.153542	46.723692
EBG	N5	124.095334	46.715634
	W5	124.072382	46.741165

### 2.2 Methodology for collecting water and sediment samples

Water samples were obtained in June 2021 using an automated sampler (W2BC-9600, Henan Mu Xiang Experimental Equipment Co., Ltd.) in accordance with GB/T 14581−93. At each of the five sampling points, three replicate samples of 5 000 mL each were collected. Sediment samples were collected in June 2021 using a mud collector (CN-100, Guangzhou Ruibin Technology Co., Ltd.). At each location, three replicate grabs were taken, yielding 500 g of sediment per replicate.

### 2.3 Water quality analysis methodology

#### 2.3.1 Method for analyzing physical and chemical indices.

The water and sediment samples were analyzed for several physical and chemical parameters to determine the quality of water. These parameters include water temperature (WT), pH, total phosphorus (TP), total nitrogen (TN), dissolved oxygen (DO), permanganate index (CODMn), ammonia nitrogen (NH3-N), Secchi depth (SD), Chlorophyll-a (Chl-a) and nitrate nitrogen (NO3-N). All indicators are measured according to the national standard method GBT5750−2023.

#### 2.3.2 Analysis of the eutrophication index.

This study used the trophic level index (TLI) to assess the eutrophication of water samples [29]. The trophic state index is computed by utilizing a specific formula:


TLI(Chl−a)=10(2.5+1.086ln(Chl−a)) (1)

TLI(TP)=10(9.436+1.624ln(TP)) (2)

TLI(TN)=10(5.453+1.694ln(TN)) (3)

TLI(SD)=10(5.118−1.94ln(SD)) (4)

$$TLI(COD_{Mn})=10(0\mathrm{.109}+2\mathrm{.661}ln(COD_{Mn}))$$ (5)

$$TLI(\sum )=\sum {\mathrm{\backslash nolimits}}_{j=\text{1}}^{m}Wj\times TLI\left(j\right)$$ (6)

Chl.a is measured in mg/m3 and SD is measured in m. All other indicators are measured in mg/L. The trophic status partition criterion is based on the TLI scores. The range of Oligotropher is TLI < 30. The range of Mesotropher is 30 ≤ TLI ≤ 50. The range of light eutropher is 50 < TLI ≤ 60; Middle eutropher is 60 < TLI ≤ 70. The range of Hyper eutropher is TLI > 70.

#### 2.3.3 Analysis of water quality index.

The water quality index is determined by the utilization of the weighted arithmetic mean technique, where each parameter is allocated a certain weight depending on its importance in assessing the total water quality. The weights were derived from expert consensus and prior research on the development of the water quality index (Table 2). The water quality index was calculated using the following formula [30]:

**Table 2:** Normalized values and weights for WQI.

Water quality indicators	Unit	Weight (pi)	Normalization factor (Ci)										
			100	90	80	70	60	50	40	30	20	10	0
pH	–	1	7	7-8	7-8.5	7-9	6.5-7	6-9.5	5-10	4-11	3-12	2-13	1-14
EC	μS/cm	1	<750	<1000	<1250	<1500	<2000	<2500	<3000	<5000	<8000	<12000	>12000
TN	mg/L	2	<0.1	<0.2	<0.35	<0.5	<0.75	<1	<1.25	<1.5	<1.75	≤2	>2
NH3-N	mg/L	3	<0.01	<0.05	<0.1	<0.2	<0.3	<0.4	<0.5	<0.75	<1	≤1.25	>1.25
TP	mg/L	1	<0.01	<0.02	<0.05	<0.1	<0.15	<0.2	<0.25	<0.3	<0.35	≤0.4	>0.4
CODMN	mg/L	3	<1	<2	<3	<4	<5	<8	<10	<12	<14	≤15	>15


$$WQI=\frac{\sum {\mathrm{\backslash nolimits}}_{i=\text{1}}^{n}CiPi}{\sum {\mathrm{\backslash nolimits}}_{i=\text{1}}^{n}Pi}$$ (7)

Where WQI is the water quality index, Pi is the weight assigned to each parameter, and Ci is the measured value of each parameter.

The Water quality is based on the WQI scores. The range of Bad is WQI < 25. The range of Low is 25 ≤ WQI ≤ 50. The range of Moderate is 50 < WQI ≤ 70; Good is 70 < WQI ≤ 90. The range of Excellent is WQI > 90.

#### 2.3.4 Analysis of integrated water quality index.

The IWQI provides a comprehensive assessment of a water body’s pollution degree and overall quality status. The IWQI model was first proposed by Mukate et al. [13] to integrate multiple water‐quality indicators into a single value for rapid evaluation of pollution levels and potability. In this study, three key parameters—NH₃–N, TN, and TP—were selected for IWQI calculation based on their dominant influence on eutrophication and overall water‐quality variability, and the assignment criteria were refined accordingly (Table 3). The formula used to calculate the IWQI is as follows:

**Table 3:** Normalized values and weights for IWQI.

Water quality indicators	Unit	Weight (pi)	Normalization factor (Ci)										
			100	90	80	70	60	50	40	30	20	10	0
TN	mg/L	2	<0.2	<0.5	<1	<1.5	<2	<3	<5	<7	<10	≤15	>15
NH3 – N	mg/L	1	<0.15	<0.5	<1	<1.1	<1.2	<1.4	<1.6	<1.8	<2	≤5	>5
TP	mg/L	1	<0.02	<0.1	<0.2	<0.22 5	<0.25	<0.27 5	<0.3	<0.35	<0.4	≤0.5	>0.5


$$IWQI=\frac{\sum {\mathrm{\backslash nolimits}}_{i=\text{1}}^{n}CiPi}{\sum {\mathrm{\backslash nolimits}}_{i=\text{1}}^{n}Pi}$$ (8)

The Water quality is based on the IWQI scores. The range of Urgent rectification is IWQI < 10. The range of Red line is 10 ≤ IWQI ≤ 20. The range of Serious pollution is 20 < IWQI ≤ 40; Moderate pollution is 40 < IWQI ≤ 60. The range of Minor pollution is 60 < IWQI ≤ 80, The range of Good is IWQI > 80.

### 2.4 Method for analyzing the organization of microbial communities

The microbial community structure was assessed using the identical methodology as described in the work conducted by Zhao et al. [31]. For microbial community analysis, two of the three 5 L duplicate water samples collected at each sampling site (total of 10 L) were pooled for DNA extraction, while the remaining 5 L duplicate sample was retained for other supplementary tests. Water DNA was isolated from 10L freshwater samples using the E.Z.N.A. Soil DNA Kit (Omega, USA), following the instructions provided by the manufacturer. DNA quality and quantity were measured with a Qubit 2.0 (Life, USA) fluorometer. The V3-V4 region of the 16S rRNA gene is amplified using a forward primer and a reverse primer. The forward primer has a sequence of CCTACGGGNGGCWGCAG, whereas the reverse primer has a sequence of GACTACHVGGGTATCTAATCC. The PCR results were analyzed by electrophoresis in 1% (w/v) agarose gels in TBE buffer (Tris, boric acid, EDTA) that are stained with ethidium bromide (EB) and seen under UV light.

### 2.5 Evaluation of the ecotoxicity

#### 2.5.1 Analysis of organic phosphorus in water and sediment samples.

Organic phosphorus compounds were quantified by ultrasonic-assisted extraction followed by gas chromatography (GC). Briefly, 10 mL of water or 10 g of wet sediment was extracted with 20 mL of acetonitrile under ultrasonication (500 W, 30 min). After centrifugation (5 000 rpm, 15 min), the supernatant was filtered through a 0.22 μm membrane and stored at 4 °C until analysis. Samples (1 μL) were injected without split into an RT-1701 capillary column (30 m × 0.25 mm × 0.25 μm). The oven program was 40 °C (hold 0 min), ramp at 30 °C/min to 130 °C, then 5 °C/min to 250 °C (hold 10 min). Carrier gas was N₂ (1.0 mL/min), with H₂ as auxiliary gas; inlet and detector temperatures were set at 270 °C and 280 °C, respectively.

#### 2.5.2 ECOSAR.

An expert tool for precisely predicting the effects of chemicals on the environment and ecotoxicity is the ECOSAR program. It is especially advantageous in assessing the potential harm that chemicals might do to aquatic plants, fish, and invertebrates. The ECOSAR model is employed in this study to evaluate the short- and long-term toxicity of organophosphorus chemicals on fish, daphnia, and green algae in Lianhuan Lake. The structural information for each compound was entered into the software by specifying its chemical name or CAS number or by uploading a molecular structure diagram, and the calculation was initiated via the software’s submit function. A chemical’s semi-lethal concentration (LC50) value, which indicates the concentration at which 50% of test organisms perish after a specific amount of time—typically 48 or 96 hours—is a quantitative measure of the chemical’s toxicity. The concentration of a chemical that has a 50% maximum influence is known as the EC50. These two measures are frequently employed to evaluate acute toxicity. The CHV refers to the concentration of a substance that induces notable harmful effects in an organism during a prolonged toxicity test. Typically, it requires extended periods of exposure to evaluate the long-term harmful effects. Taking logarithms for the three indicators LC50, EC50, and CHV helps in data analysis and comparison, according to the research results of Li et al. [32]. The anticipated toxicity value in the Globally Harmonized System (GHS) is classified using the approach outlined in Table 4. This method is used to evaluate the toxicity of organophosphorus substances.

**Table 4:** Concentration dependent toxic classification (mg/L).

Class	The Acute Toxicity	The Chronic Toxicity
Harmless	lgLC50 or lgEC50 > 0	lgCHV > 0
Harmful	−1 < lgLC50 or lgEC50 < 0	−1 < lgCHV < 0
Toxic	−2 < lgLC50 or lgEC50 < −1	−2 < lgCHV < −1
Highly toxic	lgLC50 or lgEC50 < −2	lgCHV < −2

#### 2.5.3 T.E.S.T.

The CAS numbers of each target compound were entered into the T.E.S.T. to predict fish acute toxicity (Fathead Minnow 96 h LC50) and bioconcentration factor (BCF) using the Consensus method; results were generated automatically by the software. BCF represents the ratio of the concentration of a chemical substance in an organism’s body to the concentration of the chemical substance in the environment after exposure to the environmental medium of the chemical substance (e.g., water, soil) and is used to assess the ability of a chemical substance to enrich in an organism. A high BCF value means that the chemical can accumulate in the organism and thus may pose a potential hazard to the organism and to other organisms through the food chain. The Registration, Evaluation, Authorization, and Restriction of Chemical Substances (REACH) stipulates that if the BCF value of a chemical substance is less than 2000, it means that the substance accumulates less in living organisms, is less likely to cause accumulation problems in the environment, and has less ecological risk.

#### 2.5.4 RQ.

Risk quotient assessment (RQ), originally proposed by the U.S. Environmental Protection Agency [33], is a methodology used to assess the potential risk of chemicals in the environment to ecosystems and human health (US EPA, 1989). It is calculated using the following formula:


$$RQ=\frac{MEC}{PNEC}$$ (9)

$$PNEC=\frac{LC_{50}\;OR\;EC_{50}}{AF}$$ (10)

$$RQSUM=\sum {\mathrm{\backslash nolimits}}_{i=\text{1}}^{n}RQi$$ (11)

MEC is the concentration of a chemical actually measured in an environmental sample. PNEC is the maximum concentration of a chemical substance predicted from experimental data or modeling to have no significant adverse effects on the environment and organisms. LC50 is the semi-lethal concentration, EC50 is the half-maximal effective concentration. In PNEC calculations, the value of the safety factor AF is usually based on the type and amount of toxicity data available. When acute toxicity data (e.g., EC50 and LC50) are used for PNEC calculations, a higher safety factor is usually chosen, and here AF takes the value 1000. ECOSAR provides the values for LC50 and EC50. According to the technical guide of risk evaluation prepared by the European Commission, RQ > 1 suggests high ecological risk, 0.1 < RQ < 1 suggests an intermediate ecological risk, and RQ < 0.1 suggestsa relatively low ecological risk.

## 3. Results and discussion

### 3.1 Water quality characteristics analysis

Statistical analysis of water-quality data from five sampling sites (Table 5) showed that Lianhuan Lake overall meets class V water quality (GB 3838−2002) and was mildly eutrophic. TLI values at sites W1–W5 were 54.21, 54.74, 54.66, 51.00, and 57.80, respectively, all indicating slight eutrophication; The water quality grades of sites W1, W2, and W3 were classified as Class IV, while W4 and W5 remain at Class V. WQI scores range from 42.73 to 53.64, classifying W1 and W4 as moderate quality and W2, W3, and W5 as low quality. IWQI values between 65 and 80 further confirm minor pollution at all sites. These results reflect the influence of intensive agriculture, dense population, and urban runoff in the lake’s catchment.

**Table 5:** Comprehensive water quality analysis of Lianhuan.

Sampling Number	Water quality level	TLI	Eutrophication level	WQI	Water quality	IWQI	Water quality
W1	Ⅳ	54.21	light eutropher	50.91	Moderate	80	Minor pollution
W2	Ⅳ	54.74	light eutropher	48.18	Low	77.5	Minor pollution
W3	Ⅳ	54.66	light eutropher	49.09	Low	80	Minor pollution
W4	Ⅴ	51.00	light eutropher	53.64	Moderate	77.5	Minor pollution
W5	Ⅴ	57.80	light eutropher	42.73	Low	65	Minor pollution

Consistent with other shallow-lake studies, Shi et al. [34] similarly found that Mei Lake’s water bodies were mildly eutrophic due to surrounding agricultural and anthropogenic activities. Comparable dynamics have been observed in Taihu Lake [35], where seasonal nutrient pulses drove TLI and WQI fluctuations alongside genus-level microbial shifts, and in Poyang Lake [36], where increases in TLI corresponded with marked changes in bacterial community composition. These studies underscore the sensitivity of shallow lakes to nutrient loading and the value of integrated indices (TLI, WQI, IWQI) for rapid assessment and cross-system comparisons, highlighting the need for watershed management strategies prioritizing phosphorus control and pH stabilization.

### 3.2. The analysis of Alpha diversity

Microbial diversity was assessed by calculating Shannon, Chao1, ACE and Simpson indices from Operational Taxonomic Unit (OTU) clustering results. The Shannon index integrated species richness and evenness, with higher values indicating a more diverse and evenly distributed community. The Chao1 estimator projected total species richness, with larger values reflecting greater richness. The ACE estimator, based on species-abundance data, emphasized rare species presence, so higher ACE values corresponded to increased richness and rarity. Finally, the Simpson index quantified dominance—lower Simpson values denoted higher diversity [37].

The alpha diversity index (Table 6) indicated that bacterial diversity and abundance were generally higher in sediment samples compared to water samples. In the water samples, W3 had high bacterial diversity and abundance, and the distribution of each species was relatively uniform. This may be due to the fact that agricultural activities around W3 were relatively more frequent, and under the influence of agricultural runoff, the water body may have contained higher concentrations of nutrients such as nitrogen and phosphorus, which triggered the phenomenon of eutrophication and led to the rapid reproduction of microbial populations. The higher bacterial abundance in sediments may be attributed to the fact that sediments were less influenced by water currents, offering a more stable environment, which served as a more suitable habitat for microorganisms [38]. Additionally, sediments typically contained a high concentration of organic matter (e.g., humus, plant debris) and mineral components, which supplied essential nutrients such as carbon, nitrogen, and other vital elements necessary for microbial communities. The research conducted by Li Ming’s team demonstrated that the level of species richness in the silt of the Songhua River was greater than that found in the water body [39]. The conclusion aligned closely with the findings of this investigation.

**Table 6:** Alpha Diversity index at water and sediment sampling sites.

Sample	Shannon Index	Chao1 Index	ACE Index	Simpson Index
W1	2.048	866.294	1228.489	0.27
W2	2.262	582.5	912.523	0.188
W3	4.794	2419.273	2979.378	0.029
W4	2.098	895.143	1234.312	0.256
W5	3.934	2101.944	2660.519	0.087
N1	6.284	154.797	4093.253	0.007
N2	6.341	4153.093	4135.809	0.008
N3	6.478	4045.18	4179.994	0.004
N4	6.338	4173.954	4175.893	0.006
N5	6.313	4407.745	4395.509	0.007

### 3.3. Analysis using Venn

**Fig 2:**
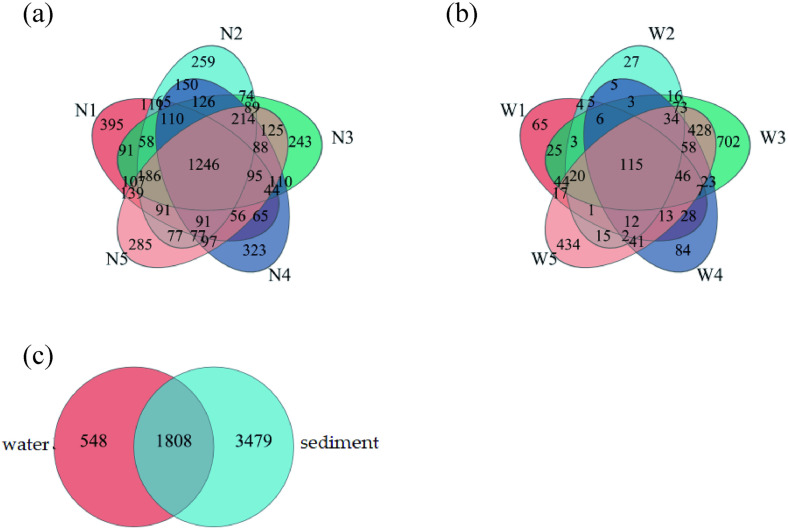
Venn diagram of sample OTU distributions. **(a)** Sediment samples distribution; **(b)** Water samples distribution; **(c)** Water and sediment samples distribution.

The Venn results (Fig 2) described the distribution of water samples, sediment samples, and water and sediment samples OTU distributions.

Fig 2 showed that bacterial species richness in the sediments was significantly higher than in water, with 1246 OTUs unique to sediments, 1808 OTUs shared by both habitats, and only 115 OTUs unique to water. The numbers of OTUs in sediment samples N1–N5 were 2950, 3024, 3006, 2957, and 3063, respectively; the number of OTUs common to all five sediment samples was 1246, accounting for 23.57% of the sediment OTUs. This was attributed to the fact that sediments offered a wider variety of substrates for microbial growth, including organic matter, minerals, and nutrients [40]. The numbers of OTUs in water samples W1–W5 were 411, 341, 1603, 482, and 1353, respectively; the number of OTUs common to all five water samples was 115, representing 4.88% of the water OTUs. The microbial diversity in the water samples from monitoring sites W3 and W5 was greater, and the similarity between the two sites was also higher. The high environmental heterogeneity of lakes provides many habitats for bacteria. The OTU number of water sample and sediment were 2356 and 5287, the number of OTU shared by water and sediment samples was 1808. There were great differences between water environment and sediment environment. Bacteria in water were more affected by river flow than sediment bacteria, and bacteria in water had greater migration rate and fusion rate. With the seasonal or hydrological period transition, the bacterial community structure also changed in the lakes, which was not only related to the turnover of the bacteria themselves, but also involved seasonal differences in environmental factors. The high number of species shared between sediment and water samples may be due to the exchange and migration of microorganisms between the water column and sediments. Wu et al found that the number of OTUs in Xiangjiang River sediment samples was higher than water samples, this conclusion was consistent with the results of this investigation [41].

### 3.4 Analysis of microbial community composition

**Fig 3:**
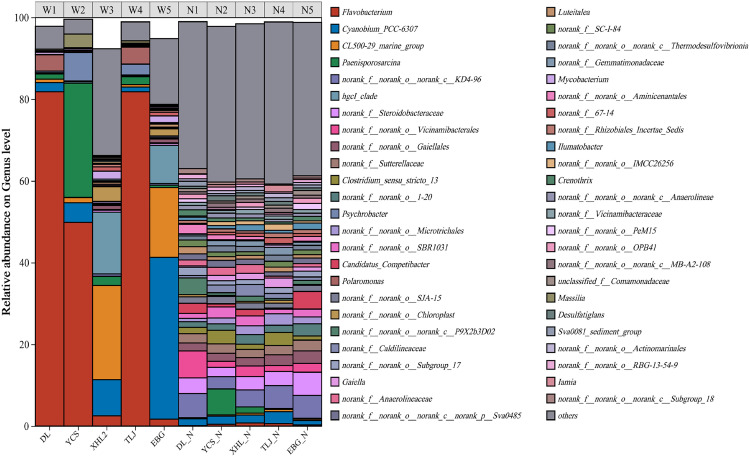
Microbial community structure at the genus level. (abundance greater than 1%).

The structure of the microbial community at the genus level (abundance greater than 1%) in Lianhuan Lake (Fig 3) indicated that the body and sediment of the lake water were composed of 1062 bacterial genera, including 716 bacterial genera in the water body and 994 bacterial genera in the sediment. The top five genera with high abundance in the water were *Flavobacterium*, *Paenisporosarcina*, *Cyanobium_PCC-6307*, *hgcI_clade* and *Psychrobacter*. The top four genera with high abundance in the sediment were *norank_f__norank_o__Vicinamibacterales*, *norank_f__norank_o__norank_c__KD4–96*, *Paenisporosarcina* and *norank_f__Steroidobacteraceae*.

The primary bacterial genera and their respective abundances in W1 were *Flavobacterium* (81.8%), *Polaromonas* (4.0%), *Cyanobium_PCC-6307* (2.2%) and *Pseudomonas* (1.9%). W2 were *Flavobacterium* (49.8%), *Paenisporosarcina* (27.9%), *Psychrobacter* (6.9%) and *Cyanobium_PCC-6307* (4.8%). W3 were *CL500–29_marine_group* (23.1%), *hgcI_clade* (15.1%), *Cyanobium_PCC-6307* (8.9%) and *norank_f__norank_o__Chloroplast* (3.6%). W4 were *Flavobacterium* (81.8%), *Polaromonas* (4.1%), *Psychrobacter* (2.6%) and *Paenisporosarcina* (2.0%). W5 were *Cyanobium_PCC-6307* (39.6%), *CL500–29_marine_group* (17.1%), *hgcI_clade* (9.3%) and *norank_f__norank_o__Chloroplast* (1.7%).

The primary bacterial genera and their respective abundances in N1 were *norank_f__norank_o__Vicinamibacterales* (6.6%), *norank_f__norank_o__norank_c__KD4–96* (5.9%), *norank_f__norank_o__norank_c__P9X2b3D02* (4.1%) and *norank_f__Steroidobacteraceae* (3.8%). N2 were P*aenisporosarcina* (6.3%), *Clostridium_sensu_stricto_13* (3.3%), *norank_f__norank_o__norank_c__KD4–96* (3.0%) and *norank_f__norank_o__SBR1031* (0.27%). N3 were *norank_f__norank_o__norank_c__KD4–96* (4.2%), *norank_f__Steroidobacteraceae* (3.3%), *norank_f__Caldilineaceae* (2.8%) and *norank_f__norank_o__Vicinamibacterales* (2.6%). N4 were *norank_f__norank_o__norank_c__KD4–96* (5.6%), *norank_f__Steroidobacteraceae* (3.5%), *Clostridium_sensu_stricto_13* (3.2%) and *Cyanobium_PCC-6307* (2.9%). N5 were *norank_f__Steroidobacteraceae* (5.7%), *norank_f__norank_o__norank_c__KD4–96* (5.6%), *Candoatus_Competibacter* (4.3%) and *norank_f__norank_o__1–20* (3.0%).

The study found that Flavobacterium, Pseudomonas and Mycobacterium and other potential pathogenic microorganisms exist in the body and sediment of the interlocking lake water. Flavobacterium is a group gram-negative bacteria and is a conditionally pathogenic bacteria, can cause pneumonia, can also incur meningitis, septicemia and other infections, the pathogenicity is not strong, generally do not cause infections, but in the body’s immunity decline may cause infections. Pseudomonas is also a gram-negative bacteria, of which Pseudomonas aeruginosa is an important human and animal pathogen that is widespread in soil, water, and hospital environments, and possesses a wide range of virulence mechanisms capable of causing a wide range of serious infections [42,43]. Mycobacterium is a specialized group of Gram-positive bacteria, of which Mycobacterium tuberculosis is the causative agent of tuberculosis, which is primarily airborne and affects the lungs [44]. Although multiple potentially pathogenic bacteria have been detected in Lianhuan Lake, whether the concentration of these bacteria can lead to the occurrence of disease needs further study.

Bacteria associated with phosphorus metabolism were found in abundance in both the water column and sediments of Lianhuan Lake, including Flavobacterium, Pseudomonas, Candoatus_ Solibacter, Massilia. The species of these microorganisms were abundant and relatively high, probably because the Lianhuan Lake has been in a mild eutrophic state, which promoted the phosphorus metabolic activity of the microorganisms in the lake [45]. Other scholars have also found that a large number of phosphorus metabolizing microorganisms such as Pseudomonas, Bacillus, Azotobacter, Acinetobacter, Rhodobacter, etc. were present in the lake reservoirs [46].

### 3.5 Analysis of the correlation between environmental variables and microorganisms

**Fig 4:**
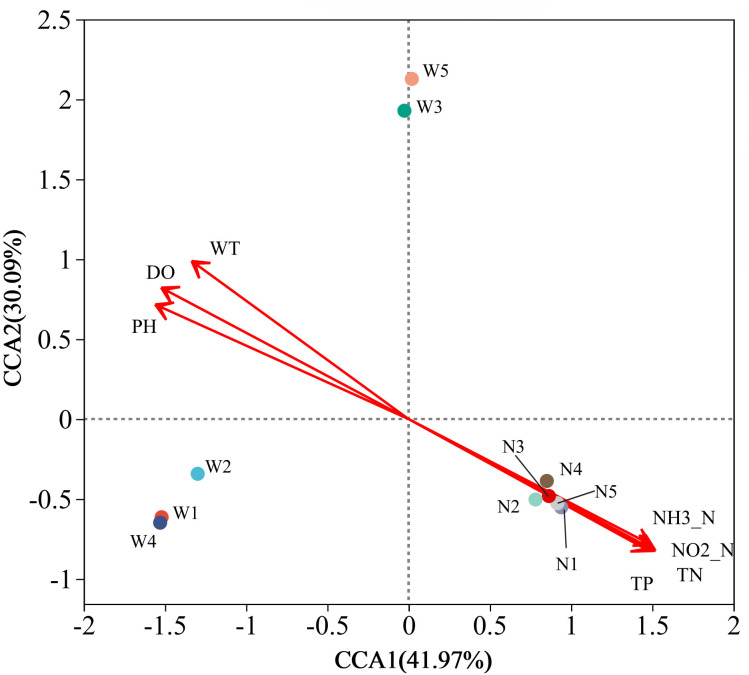
CCA analysis at the genus level.

The correlation between environmental variables and microorganisms (Fig 4) showed that the first two axes jointly explained 72.06% of the variance. Factors were ranked by their absolute loadings on CCA1—which explained the largest share of variance—so that longer projections on this axis indicated stronger influence; the order of influence of environmental factors on bacterial community structure was pH > TP > DO > TN > NH₃–N > NO₂–N > WT. The pH level had the greatest impact on water samples. This is because most aquatic microbes thrive in conditions that are neutral or slightly alkaline. However, excessive pH values, either too acidic or too alkaline, could hinder the development and metabolism of microorganisms and disrupt their activities. TP had the second highest impact. Phosphorus-metabolizing microorganisms relied on enzymes such as phosphatases for phosphorus metabolism, and the activity of these enzymes was highly dependent on the pH of the environment. Enzyme activity is highest in the optimal pH environment, which promoted the efficient utilization and conversion of phosphorus [47]. W1, W2, W3 and W4 were positively correlated with WT, DO and pH. N1, N2, N3, N4, N5 and W5 were positively correlated with TP, NH₃–N, NO₂–N and TN. To further analyze organic phosphorus in lakes and conduct ecological safety evaluations had important implications. In general, the effect of phosphorus on the bacterial community structure was more significant, the terrain in northern China is flat, the soil is fertile, per-capita arable land was higher, agricultural cultivation was more developed, and the main source of phosphorus pollution in shallow lakes was the application of pesticides and fertilizers; further analysis of the distribution of organic phosphorus in Lianhuan Lake and its ecological security was very necessary.

### 3.6 Organophosphorus ecological safety analysis

#### 3.6.1 Analysis of the organophosphorus content.

**Fig 5:**
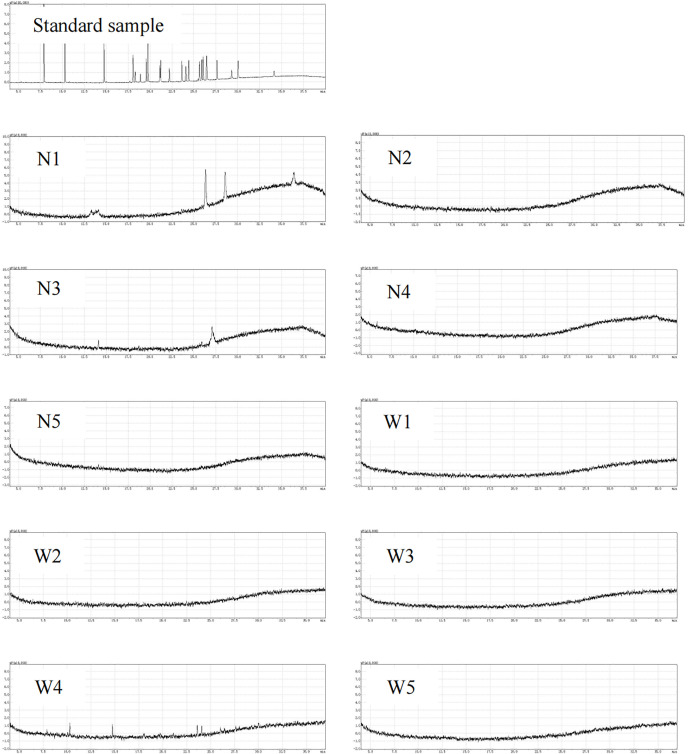
Analysis of organic phosphorus in sediment and water samples.

The results of the organic phosphorus gas analysis (Fig 5 and Table 7) of the water bodies and sediments of the Lianhuan Lake showed that A total of ten organophosphates are detected, Thionazin (0.010 µg/L), Ethoprophos (0.013 µg/L in N1, 0.028 µg/L in N3), Suprofos (0.161 µg/L in N1, 0.075 µg/L in N3), EPN (0.246 µg/L), Coumaphos (0.101 µg/L), O,O,O-Triethylphosphorothioate (0.008 µg/L), Mevinphos (0.015 µg/L), Naled (0.021 µg/L), Z-Tetrachlorvinphos (0.043 µg/L) and Prothiophos (0.019 µg/L). Organophosphorus compounds in N1 included thionazin, ethoprophos, sulprofos, EPN and coumaphos, with a concentration of 0.010, 0.013, 0.161, 0.246 and 0.101 µg/L, respectively. Thionazin was an organophosphorus pesticide that was mainly used to control pests on crops. Ethoprophos was a broad-spectrum agent for controlling nematodes and underground pests. Sulprofos killed microorganisms and insects in water and was often used as an insecticide and fungicide. EPN was widely applied in controlling various pests, and it could also slow down the burning rate. Coumaphos was a non-endogenous insecticide, particularly effective against Diptera pests, and was also used to control ectoparasites, with remarkable effect on skin flies. Organophosphorus compounds in N3 included ethoprophos and sulprofos, with concentrations of 0.028 and 0.075 µg/L, respectively. Organophosphorus compounds in W4 included O,O,O-Triethylphosphorothioate, mevinphos, naled, Z-Tetrachlorvinphos and prothiophos, with concentrations of 0.008, 0.015, 0.021, 0.053 and 0.019 µg/L, respectively. O,O,O-Triethylphosphorothioate was mainly used as an insecticide and could effectively control various pests. Mevinphos was an organophosphorus insecticide that inhibited acetylcholinesterase and had broad-spectrum insecticidal action, commonly used against chewing and sucking insects and leaf mites. Naled was an organophosphorus insecticide produced by the reaction of dichlorvos with bromine; it had a wide activity spectrum, including contact, stomach toxicity and fumigation. Z-Tetrachlorvinphos was a highly effective and low-toxic organophosphorus insecticide; it was highly effective against Lepidoptera, Diptera and various Coleoptera pests, but had low toxicity to warm-blooded animals. Prothiophos was frequently utilized in agricultural fields and orchards for managing and preventing pests and diseases. Furthermore, it could be used in both residential and industrial settings for insecticidal and antiseptic purposes Organophosphorus compounds in N3 included ethoprophos and sulprofos, with concentrations of 0.028 and 0.075 µg/L, respectively. Organophosphorus compounds in W4 included O,O,O-Triethylphosphorothioate, mevinphos, naled, Z-Tetrachlorvinphos and prothiophos, with concentrations of 0.008, 0.015, 0.021, 0.053 and 0.019 µg/L, respectively. O,O,O-Triethylphosphorothioate was mainly used as an insecticide and could effectively control various pests. Mevinphos was an organophosphorus insecticide that inhibited acetylcholinesterase and had broad-spectrum insecticidal action, commonly used against chewing and sucking insects and leaf mites. Naled was an organophosphorus insecticide produced by the reaction of dichlorvos with bromine; it had a wide activity spectrum, including contact, stomach toxicity and fumigation. Z-Tetrachlorvinphos was a highly effective and low-toxic organophosphorus insecticide; it was highly effective against Lepidoptera, Diptera and various Coleoptera pests, but had low toxicity to warm-blooded animals. Prothiophos was frequently utilized in agricultural fields and orchards for managing and preventing pests and diseases. Furthermore, it could be used in both residential and industrial settings for insecticidal and antiseptic purposes. Approximately 362,000 acres of agricultural land were located around Lianhuan Lake, of which 237,000 acres were planted to corn; 87,000 acres to rice, 33,000 acres to soybeans, and 3,000 acres to potatoes. As a result of extensive agricultural activity, pesticides (especially organophosphate pesticides) were heavily used to control pests and diseases. These pesticides entered the lake through surface runoff, irrigation drainage, and rainfall washout, resulting in increased concentrations of organophosphorus compounds in the water column.

**Table 7:** Organic phosphine components from Lianhuan Lake.

Samples	Organic Phosphonic	Chemical Formula	Organophosphorus Concentration(µg/L)	CAS
N1	Thionazin	C8H13N2O3PS	0.010	297-97-2
	Ethoprophos	C8H19O2PS2	0.013	13194-48-4
	Suprofos	C12H19O2PS3	0.161	35400-43-2
	EPN	C14H14NO4PS	0.246	3878-45-3
	Coumaphos	C14H16ClO5PS	0.101	56-72-4
N3	Ethoprophos	C8H19O2PS2	0.028	13194-48-4
	Suprofos	C12H19O2PS3	0.075	35400-43-2
W4	O,O,O-Triethylphosphorothioate	C6H15O3PS	0.008	126-68-1
	Mevinphos	C7H13O6P	0.015	7786-34-7
	Naled	C4H7Br2Cl2O4P	0.021	300-76-5
	Z-Tetrachlorvinphos	C10H9Cl4O4P	0.043	22248-79-9
	Prothiophos	C11H15Cl2O2PS2	0.019	34643-46-4

#### 3.6.2 ECOSAR.

**Fig 6:**
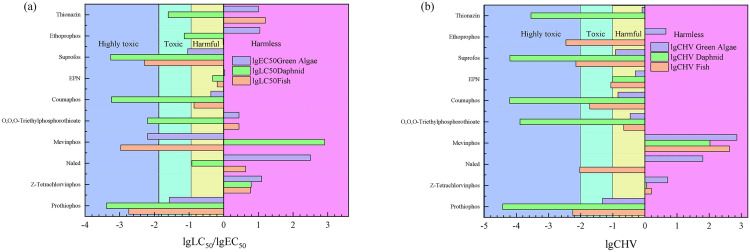
Analysis of acute and chronic toxicity of organophosphorus. **(a)** The acute toxicity; **(b)** The chronic toxicity.

The results of the ECOSAR evaluation of the toxicity of organophosphorus to aquatic plants, fish and invertebrates (Fig 6) indicated that in acute and chronic toxicity assessments, nine organophosphorus compounds—Thionazin, Ethoprophos, Suprofos, EPN, Coumaphos, O,O,O-Triethylphosphorothioate, Mevinphos, Naled, and Prothiophos—were found to present different levels of risk to fish, Daphnia and algae. Suprofos, Coumaphos and Prothiophos were highly toxic to Daphnia, and Suprofos and Prothiophos were highly toxic to fish. In acute toxicity studies, Thionazin was found to be toxic to daphnid, harmless to fish and green algae. Ethoprophos was toxic to daphnid, harmful to fish and harmless to green algae. Suprofos was highly toxic to daphnid and fish, toxic to green algae. EPN was toxic to daphnid and fish, harmless to green algae. Coumaphos was highly toxic to daphnid and toxic to fish and green algae. O,O,O-Triethylphosphorothioates was highly toxic to daphnid and harmless to fish and green algae. Mevinphos was highly toxic to fish and green algae, harmful to daphnid. Naled was toxic to daphnid and harmless to fish and green algae. Z-Tetrachlorvinphos was harmless to daphnid, fish and green algae. Prothiophos was highly toxic to daphnid and fish, toxic to green algae. In chronic toxicity studies, Thionazin was found to be highly toxic to daphnid, harmful to green algae and harmless to fish. Ethoprophos was highly toxic to fish, harmless to daphnid and green algae. Suprofos was highly toxic to daphnid and fish, harmful to green algae. EPN was toxic to daphnid and fish, harmless to green algae. Coumaphos was highly toxic to daphnid, toxic to fish, and harmful to green algae. O,O,O-Triethylphosphorothioates was highly toxic to daphnid, toxic to fish and harmful to green algae. Mevinphos was harmless to daphnid, fish and green algae. Naled was highly toxic to fish and harmless to daphnid and green algae. Z-Tetrachlorvinphos was harmless to daphnid, fish and green algae. Prothiophos was highly toxic to daphnid and fish, toxic to green algae.

Both Suprofos, Coumaphos and Prothiophos belonged to the class of organophosphate esters, which interfere with nervous system function by inhibiting acetylcholinesterase (AChE) activity. Since these compounds had similar toxicity mechanisms in aquatic organisms, they exhibited high toxicity to invertebrates such as Daphnia [48]. Therefore, the amount and frequency of use of organophosphorus pesticides should be reasonably controlled to protect the ecological safety of Lianhuan Lake.

#### 3.6.3 T.E.S.T.

The results of the evaluation of the BCF and the LC₅₀ in Lianhuan Lake (Table 8) showed that the BCF values for the organophosphorus compounds in all samples were less than 2000, indicating that these organophosphorus compounds had a relatively limited tendency to accumulate in organisms and a low risk of transmission through the food chain. None of the organophosphorus concentrations detected reached the lethal endpoint concentration when compared to the 96-hour LC₅₀ for fathead minnow automatically generated by the T.E.S.T. software.

**Table 8:** BCF and LC50 prediction of organophosphorus components (mg/L).

Samples No.	Organophosphonic	BCF	LC50 (mg/L)	Organophosphorus Concentration(µg/L)	Over LC50 Yes or No
N1	Thionazin	39.03	7.95	0.010	No
	Ethoprophos	33.49	0.95	0.013	No
	Suprofos	251.06	0.35	0.161	No
	EPN	378.27	2.46	0.246	No
	Coumaphos	/	0.95	0.101	No
N3	Ethoprophos	33.49	0.95	0.028	No
	Suprofos	251.06	0.35	0.075	No
W4	O,O,O-Triethylphosphorothioate	7.57	35	0.008	No
	Mevinphos	/	48.67	0.015	No
	Naled	5.90	4.19	0.021	No
	Z-Tetrachlorvinphos	598.54	0.17	0.043	No
	Prothiophos	354.29	0.0983	0.019	No

Note:/ indicates that it is not queried in T.E.S.T

#### 3.6.4 RQ.

The RQ assessment results (Table 9) showed that, in high ecological risk studies, Suprofos posed a high ecological risk to fish, daphnid, and green algae; Coumaphos posed a high ecological risk to daphnid; O,O,O-Triethylphosphorothioate posed a high ecological risk to daphnid; mevinphos posed a high ecological risk to fish and green algae; and Prothiophos posed a high ecological risk to fish and daphnid. Suprofos was a highly toxic organophosphorus compound. Suprofos was harmful to humans in that it caused symptoms of poisoning such as headache, dizziness, nausea and vomiting. High doses or prolonged exposure may cause serious effects on the nervous system, liver and kidneys [49]. The danger of Mevinphos to the human body was manifested in its ability to inhibit cholinesterase activity. It caused dizziness, weakness, vomiting, excessive sweating, salivation, and in severe cases, muscle spasms, coma, respiratory distress, and pulmonary edema. Prothiophos was highly toxic to aquatic organisms and had long-lasting effects [50]. The study on moderate ecological risks found that Thionazin posed moderate ecological risks to daphnid; Ethoprophos posed moderate ecological risks to daphnid; EPN posed moderate ecological risks to fish, daphnid, and green algae; Coumaphos posed moderate ecological risks to fish and green algae; Naled posed moderate ecological risks to daphnid; and Prothiophos posed moderate ecological risks to green algae. Ethoprophos and Suprofos were detected in both N1 and N3, and although their concentrations were different, the ecological risk trends were the same.

**Table 9:** RQ calculations for organophosphorus components.

Samples NO.	Organic Phosphonic	RQ		
		Fish	Daphnid	Green Algae
N1	Thionazin	0.001	0.4	0.001
	Ethoprophos	0.013	0.174	0.001
	Suprofos	31.01	293.2	1.771
	EPN	0.378	0.511	0.23
	Coumaphos	0.739	177.6	0.236
N3	Ethoprophos	0.029	0.381	0.002
	Suprofos	14.42	136.3	0.824
W4	O,O,O-Triethylphosphorothioate	0.002	1.19	0.002
	Mevinphos	13.89	0	2.369
	Naled	0.005	0.175	0
	Z-Tetrachlorvinphos	0.007	0.007	0.003
	Prothiophos	10.41	44.64	0.694

In summary, based on the results of the three evaluations of ECOSAR, T.E.S.T. and RQ, the organophosphorus compounds detected in Lianhuan Lake—Suprofos, Coumaphos and Prothiophos—were highly toxic to daphnia, and Suprofos and Prothiophos were highly toxic to fish, but at concentrations below the LC₅₀ for fish. Suprofos, mevinphos and Prothiophos had high ecological risk. In order to reduce the harm of organophosphorus pesticides to water ecology, it is recommended to apply organophosphorus pesticides scientifically, encourage the use of biopesticides, avoid overdose, and reduce residues.

## 4. Conclusions

Shallow lakes are ecologically important for maintaining biodiversity, regulating hydrological processes, purifying water, and supporting ecosystem services. This study selected Lianhuan Lake—a typical submersible lake in northern China—as the research subject. We analyzed its water‐quality characteristics, examined bacterial diversity in the water column and sediments, assessed organophosphorus pesticide pollution, and evaluated ecological safety. The results showed that the lake’s water quality meets Class V standards (GB 3838−2002), indicating only mild eutrophication (TLI) and minor contamination (WQI, IWQI). Across water and sediment samples, we identified 59 phyla, 180 classes, 407 orders, 520 families, and 1 062 genera, with diversity consistently higher in sediments. Water‐quality indicators influenced bacterial diversity in the order pH > TP > DO > TN > NH₃-N > NO₂-N > WT. Gas‐chromatography detected ten organophosphorus compounds in samples N1, N3, and W4: thionazin, ethoprophos, suprofos, EPN and coumaphos (N1); ethoprophos and suprofos (N3); and O,O,O-triethylphosphorothioate, mevinphos, naled, Z-tetrachlorvinphos and prothiophos (W4). ECOSAR results indicated that nine compounds exhibited varying degrees of acute and chronic toxicity to fish, Daphnia, and algae, with suprofos, coumaphos, and prothiophos highly toxic to Daphnia and suprofos and prothiophos highly toxic to fish (though at concentrations below the fish LC₅₀). T.E.S.T. predictions showed that neither BCF nor LC₅₀ values exceeded regulatory thresholds, while RQ assessment identified suprofos, mevinphos, and prothiophos as high ecological‐risk compounds. This work delivers the first integrated baseline assessment of Lianhuan Lake’s physicochemical status, microbial community structure, and organophosphorus pesticide–derived ecological risks, providing critical data and targeted recommendations for its sustainable management and conservation.
